# Configuration and Design of Electromagnets for Rapid and Precise Manipulation of Magnetic Beads in Biosensing Applications

**DOI:** 10.3390/mi10110784

**Published:** 2019-11-15

**Authors:** Moshe Stern, Meir Cohen, Amos Danielli

**Affiliations:** Faculty of Engineering, Bar-Ilan University, Ramat Gan 5290002, Israel; moshikst1@gmail.com (M.S.); meircohen486@gmail.com (M.C.)

**Keywords:** magnetic tweezers, magnetic beads, biosensing, pole tip, Zika virus

## Abstract

Rapid and precise manipulation of magnetic beads on the nano and micro scales is essential in many biosensing applications, such as separating target molecules from background molecules and detecting specific proteins and DNA sequences in plasma. Accurately moving magnetic beads back and forth requires at least two adjustable magnetic field gradients. Unlike permanent magnets, electromagnets are easy to design and can produce strong and adjustable magnetic field gradients without mechanical motion, making them desirable for use in robust and safe medical devices. However, using multiple magnetic field sources to manipulate magnetic beads presents several challenges, including overlapping magnetic fields, added bulk, increased cost, and reduced durability. Here, we provide a thorough analysis, including analytical calculations, numerical simulations, and experimental measurements, of using two electromagnets to manipulate magnetic beads inside a miniature glass cell. We analyze and experimentally demonstrate different aspects of the electromagnets’ design, such as their mutual influence, the advantages and disadvantages of different pole tip geometries, and the correlation between the electromagnets’ positions and the beads’ aggregation during movement. Finally, we have devised a protocol to maximize the magnetic forces acting on magnetic beads in a two-electromagnet setup while minimizing the electromagnets’ size. We used two such electromagnets in a small footprint magnetic modulation biosensing system and detected as little as 13 ng/L of recombinant Zika virus antibodies, which enables detection of Zika IgM antibodies as early as 5 days and as late as 180 days post symptoms onset, significantly extending the number of days that the antibodies are detectable.

## 1. Introduction

Small spherical magnetic beads—usually consisting of a paramagnetic core embedded in a non-magnetic matrix, such as a polymer or quartz [1,2]—are widely used in bioanalysis and medical applications [2,3,4,5,6]. These applications include separating target molecules from background molecules [7,8,9], investigating molecular interactions inside living cells [5,10,11], directing neuronal migration and growth [12], detecting specific proteins and DNA sequences in plasma [13,14,15,16], and reducing background in opto-chemical sensing [17]. Many of these applications require accurate manipulation of the magnetic beads on the nano and micro scales. For example, Anker et al. (2003) developed magnetically modulated optical nanoprobes (MagMOONs) [17,18] that blink in response to rotating magnetic fields. Separating the signal of the blinking probe from the unmodulated background enables simple and sensitive detection of target analytes at low concentrations. Danielli et al. (2008) used a magnetic modulating biosensing (MMB) system to detect proteins and specific DNA sequences. In MMB, the target molecules are captured by fluorescently labeled magnetic beads that are aggregated and manipulated in and out of an excitation beam, separating their signal from that of Raman scattering of water molecules and unbound fluorescently labeled probes [13,14,15,16].

To remotely manipulate magnetic beads, an external magnetic field is applied. A constant magnetic field can rotate the magnetic beads, and a magnetic field gradient exerts a force on the beads that can displace them. The design of external magnetic field sources affects the forces acting on the magnetic beads. In applications that involve separation of a target molecule from background molecules, a single magnetic field gradient source, e.g., a permanent magnet, is used. The gradient pulls the magnetic beads, which are attached to the target molecules, onto the sample holder’s wall, and the rest of the sample is then washed out. However, a single magnet can only pull the magnetic beads, but not push them away [1]. Therefore, when precise manipulation of beads in space is required, several magnets are used. To manipulate magnetic beads along a straight line, in a plane, or in space, at least two, three, or six magnets are required, respectively [5,19]. The use of multiple magnetic field sources to manipulate magnetic beads presents several challenges, including overlapping magnetic fields, added bulk, increased cost, and reduced durability. In many lab-on-a-chip applications, permanent magnets are the most accessible source of a strong magnetic field [12,20]. However, to adjust the field strength and manipulate magnetic beads, permanent magnets have to be physically rotated or translated [17]. Alternatively, electromagnets, which convert electrical current into a magnetic field and allow rapid control of the field strength, can be turned on or off without mechanical movement [1,21] and thereby have less risk of malfunction. The simplest version of an electromagnet is a coiled conductive wire. Multiple coils that are formed in a cylindrical geometry construct a solenoid. When electrical current runs through the wire, a magnetic field is established in the direction of the solenoid’s axis. The magnetic field is concentrated into a nearly uniform field in the center of the solenoid and is weaker and diverges outside of the solenoid. To amplify the magnetic field, a magnetic iron core is positioned inside the solenoid. The cores, also called poles, are typically made of a soft ferromagnetic material (e.g., iron or cobalt-iron alloys) [1]. 

The manipulation of a single magnetic bead by either a single or by several electromagnets has been extensively analyzed [1,5,15,22]. However, manipulating multiple beads by using two electromagnets has yet to be explored. Moreover, recent publications have described the particular effectiveness of electromagnets with sharpened pole tips in manipulating magnetic beads [1,5,19,21,23]. A sharp pole tip induces higher forces on beads that are near the tip. To date, only basic cone [22] and parabolic [5] pole-tip shapes have been analyzed and experimentally tested. Here, we analyze and experimentally demonstrate these and other aspects of the beads’ manipulation, including the mutual influence of electromagnets, the advantages and disadvantages of different pole tip geometries, and the correlation between the position of the electromagnets and the beads’ aggregation during movement. Finally, we have devised a protocol to maximize the magnetic forces acting on magnetic beads in a two-electromagnet setup while minimizing the electromagnets’ size and power consumption. Using two electromagnets in a small footprint magnetic modulation biosensing system, we detected as little as 13 ng/L of recombinant Zika virus (ZIKV) antibodies, which enables detection of Zika IgM antibodies as early as 5 days and as late as 180 days post symptoms onset, significantly extending the number of days that the antibodies are detectable. [16].

## 2. Materials and Methods

### 2.1. Evaluating Different Pole Tip Shapes

**Figure 1:**
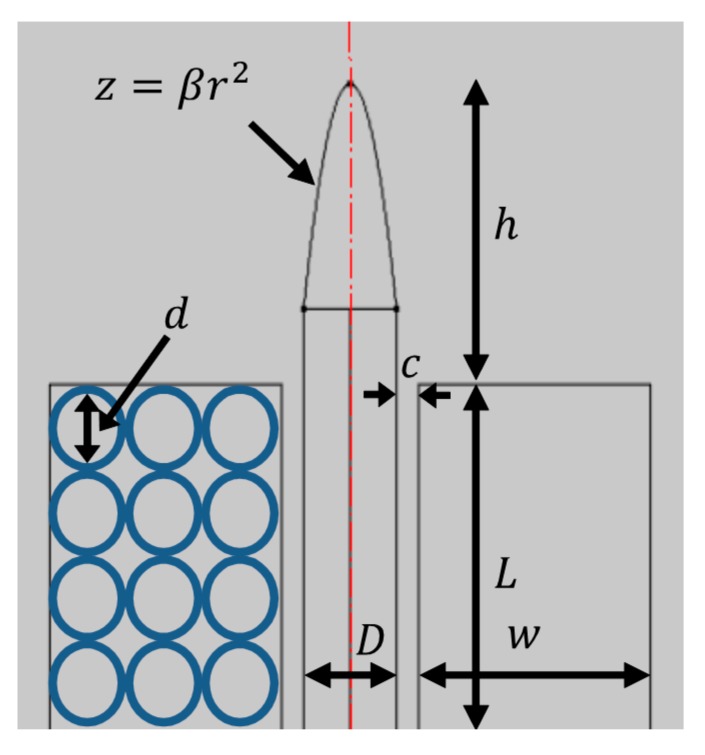
The geometry of an electromagnet with a parabolic pole tip. L and w, length and width of the windings area; d, wire diameter; D, magnetic pole diameter; c, core sleeve’s width; *β*, parabolic curvature of the pole tip; and h, the distance from the coil edge to the pole’s tip.

To evaluate different shapes of magnetic pole tips, we used a single electromagnet setup. In Figure 1, the electromagnet’s mechanical dimensions are denoted by L, the length of the coil; w, the total winding area thickness; h, the distance from the coil to the pole’s tip (e.g., a parabolic pole tip with a curvature coefficient, β); D, the diameter of the magnetic pole; c, the core’s sleeve width; and d, the diameter of the wire (not including its insulating coating).

**Figure 2:**
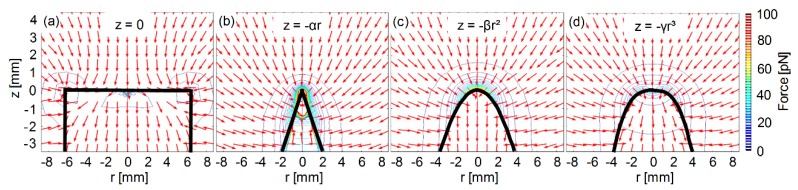
COMSOL Multiphysics® simulations of the magnetic force profiles of different pole tip shapes. The magnetic force profiles of four pole tip shapes; (**a**) flat-top, (**b**) conical, (**c**) parabolic, and (**d**) cubic. The color map of the contour lines represents the magnitude of the force, and the arrows represent the direction of the force.

We evaluated cylindrical magnetic poles (D=12.7 mm) with four different tip shapes: A flat, a cone, a paraboloid, and a cube (Figure 2). The magnetic field profile of each pole was simulated assuming an electromagnet with the following parameters: L=100 mm, w=31.7 mm, h=35 mm, d=0.75 mm (not including coating), and current I=1 A. For consistency, the tip of each magnetic pole was set at (z,r)=(0,0), and the distance from the coil edge to the pole tips was fixed at h=35 mm. To calculate the magnetic force acting on a magnetic bead as a function of the distance from the pole’s tip along the electromagnet’s symmetry axis, we considered a magnetic bead (Dynabeads M-280 Streptavidin, Thermo Fisher, Waltham, MA, USA) [24] whose magnetic moment was saturated at $m_{s}=1.8\cdot 10^{-13}$ A·m^2^.

### 2.2. Analytical and Numerical Models of the Magnetic Force Acting on a Magnetic Bead 

In general, magnetic beads’ movement in a fluid is mainly affected by the magnetic force and the drag force, $F_{drag}$, which, for a spherical particle, is given by [1,2,9]

(1) $$F_{drag}=6\pi \eta rv$$

where η is the solution viscosity (e.g., $\eta =8.9\cdot 10^{-4}\;Pa\cdot sec$ in water), r is the particle radius, and v is the particle velocity. An accurate model of the beads’ movement in solution should consider both forces [15]. However, the purpose of this paper is to explore different aspects of electromagnets’ design, which mainly affects the magnetic force acting on the beads. Hence, our simulation focused on comparing the magnetic force in space for different designs and configurations of the electromagnets.

To derive an analytical expression of the force acting on a magnetic bead, two assumptions were made. First, the bead is a single, zero-dimensional object. Second, the magnetic field surrounding the pole tip is sufficiently high that the bead’s magnetic moment is saturated. Without these assumptions, the magnetic moment of the bead becomes spatially-dependent rather than constant, and the calculation of the force is needlessly complicated. However, under these assumptions, the force acting on the bead depends simply on the bead’s saturation moment and the magnetic field gradient at the bead’s specific location. Overall, the force, $F_{m}$, acting on a magnetic bead that has a saturated magnetic moment, $m_{s}$, and is located at position z,r is given by (Supplementary Material, Appendix A):

(2) $$F_{m}=\left(m_{s}/\left|\vec{B}\right|\right){\left({\left(B_{r}\partial B_{r}/\partial r+B_{z}\partial B_{z}/\partial r\right)}^{2}+{\left(B_{r}\partial B_{r}/\partial z+B_{z}\partial B_{z}/\partial z\right)}^{2}\right)}^{1/2}$$

where $\vec{B}$ is the magnetic field, and $B_{z}$ and $B_{r}$ are the scalar components of the magnetic field in the z and r directions. Simulations using COMSOL Multiphysics® magnetic field module showed that the magnetic moment of the bead used in our setup saturates around the pole tips (Supplementary Material, Figure S1). Moreover, the bead (2.8 µm in diameter) is much smaller than the 12.7 mm diameter of the electromagnetic pole. Therefore, in our setup both assumptions are valid.

### 2.3. Manipulation of Magnetic Beads Using a Two-Electromagnet Setup

**Figure 3:**
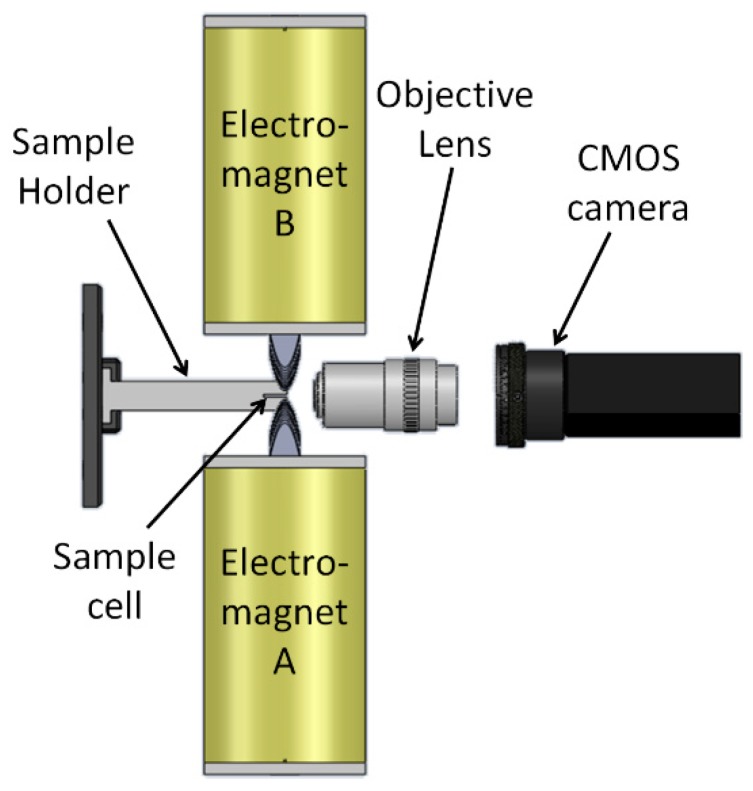
Experimental setup of two electromagnets with parabolic pole-tips that are positioned opposite each other. The sample cell is positioned between the poles using a sample holder and an XYZ translational stage. The movement of the magnetic beads in the cell is imaged to the back plane of an objective lens and visualized using a CMOS camera.

To manipulate magnetic beads back and forth along a straight line, two electromagnets that are facing each other are required. To analyze the characteristics of this manipulation, we used two identical electromagnets, labeled A and B in Figure 3, each having a parabolic pole tip (β=0.65 mm^−1^) and the following dimensions: L=100 mm, w=31.7 mm, h=35 mm, D=12.7 mm, c=3 mm, and d=0.75 mm. The number of turns was 3900, and the measured resistance was 24 ohms.

To demonstrate and analyze the movement of magnetic beads, we used ~30,000 magnetic beads (Dynabeads M-280 Streptavidin, Thermo-Fischer, Waltham, MA, USA) mixed in phosphate buffered saline with Tween-20 buffer (PBST) and placed in a rectangular bottom-sealed borosilicate sample cell whose inner dimensions were 8 mm × 0.4 mm × 70 mm (Vitrocom, 2540, Boonton, NJ, USA). The sample cell was held between the electromagnets so that the beads could be manipulated along its narrow dimension (i.e., 0.4 mm). When the two electromagnets were alternately turned on and off, the magnetic beads moved periodically between them. The movement of the magnetic beads was imaged to the back plane of an objective lens (Newport, M-10×, 0.25NA, Newport, CA, USA) and visualized using a CMOS camera (FLIR, GS3-U3-23S6M-C, Wilsonville, OR, USA). 

### 2.4. Evaluating the Effects of Mutual Magnetization in a Two-Electromagnet Setup

**Figure 4:**
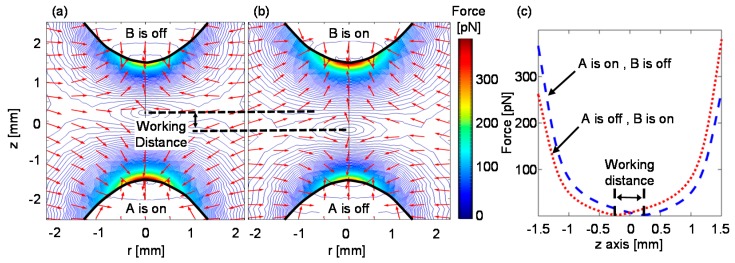
The magnetic force profile of two electromagnets positioned opposite each other. Simulated magnetic field profile when (**a**) electromagnet A (bottom) is active and electromagnet B (top) is inactive, and conversely, (**b**) when electromagnet A (bottom) is inactive and electromagnet B (top) is active. The distance between the parabolic pole tips (β=0.65 mm^−1^) is 3 mm. The contour lines represent the magnitude of the force, and the arrows represent its direction. (**c**) The working distance is defined as the distance between the positions along the z-axis, in which the magnetic force is zero.

When two or more electromagnets are used to manipulate magnetic beads, mutual magnetizing of the pole tips and the remnant magnetic fields of the ferromagnetic cores affect the forces acting on the magnetic beads. For example, in a two-electromagnet setup, when one of the electromagnets is active and the other is inactive, the beads are expected to move towards the active magnet. However, due to remanence and the influence of the active electromagnet, the ferromagnetic core of the inactive electromagnet is magnetized, and it attracts nearby beads. In Figure 4, when electromagnet A is active, the magnetic force along its symmetry axis decreases until it reaches zero at a point beyond half the distance to the inactive electromagnet B. Beyond this point, the magnetic force increases, but its direction changes towards the inactive electromagnet. Hence, although electromagnet B is inactive, beads that are in its proximity will be attracted to it rather than towards the active electromagnet A. Consequently, the distance over which the magnetic beads can be manipulated back and forth without being trapped by one of the electromagnets is smaller than the actual distance between the electromagnetic pole tips. Here, we defined this distance as the “working distance”, and it is calculated by subtracting the positions along the z-axis in which the force is zero (Figure 4c).

First, we simulated the correlation between the working distance and different parameters, such as the distance between pole tips, the pole tip’s parabolic curvature, and the electric signal profile. To correlate the working distance and the distance between the pole tips, we assumed that the parabolic curvature of the tips was β=0.65 mm^−1^. To correlate the working distance and the pole tips’ parabolic curvature, we assumed that the distance between the pole tips was 3 mm. For the correlation between the working distance and the electric signal profile, we assumed that the distance between the pole tips was 3 mm and the parabolic curvature of the tips was β= 0.25, 0.65, and 0.85 mm^−1^. In addition, we assumed that the electromagnets were activated by two square waves, with a 50% duty cycle and a 180° phase shift between them. The peaks of the square waves were 36 V, and the valleys ranged between 0 V to −36 V. Therefore, the ratio between the voltages of the electromagnets, $-V_{2}/V_{1}$, ranged from 0 to 1. For each correlation study, we calculated the magnetic force along the z-axis between the electromagnets’ pole tips and derived the working distance. 

Second, we experimentally validated the correlation between the working distance and the distance between the pole tips. We placed the magnetic poles (β=0.65 mm^−1^) 3 mm and 4 mm apart and imaged the beads’ movement inside rectangular glass cells that had various inner widths, ranging from 0.4 mm to 0.8 mm (Vitrocom, 2540, 4905, 4806, 4707, 4608). The electromagnets were activated by two square waves, with an amplitude of 36V, a 50% duty cycle, and a 180° phase shift between them. 

Third, we explored how the magnetic beads move between two electromagnets with parabolic pole tips. When the two electromagnets, positioned opposite each other, were placed in proximity, the magnetic beads moved along a narrow line on the z-axis between the pole tips. This movement resembled an hourglass with a narrow neck. To evaluate the correlation between the width of the neck and the distance between the pole tips, we placed a bottom-sealed borosilicate sample cell with an inner width of 0.6 mm (Vitrocom, 4806, Boonton, NJ, USA) between two electromagnets with parabolic pole tips (β=0.65 mm^−1^). Using XYZ translational stages, we changed the distance between the pole tips from 1 mm to 7 mm. For each distance, the beads’ movement was recorded, and the width of the neck was measured.

### 2.5. Optimizing Magnetic Forces in a Two-Electromagnet Setup

To maximize the magnetic force of an electromagnet while minimizing its physical dimensions and power consumption, we calculated the tradeoff between the winding area width, w, and the diameter of the pole, D. Based on the physical constraints of the setup, one can determine the total diameter of the electromagnet, S, which is defined as:

(3) S=D+2w+2c

where c is the core sleeve’s width. For three fixed total diameters of the electromagnet (S= 45, 50, and 55 mm), we simulated the magnetic force acting on a magnetic particle located along the symmetry axis at z= 2 mm from the parabolic pole tip as a function of the winding area width. The core sleeve’s width was assumed to be 3 mm (i.e., c= 3 mm), and the electromagnet parameters were set at L= 100 mm, h= 35 mm, d= 0.75 mm, β= 0.65 mm^−1^, and I=1 A.

### 2.6. Preparing the MMB IgM anti-Zika NS1 Dose Response 

To demonstrate the use of two optimally designed electromagnets in a biosensing application, we measured increasing concentrations of recombinant ZIKV antibodies (Human IgM anti-Zika NS1 monoclonal antibodies, MAB12123, Native Antigen) in an MMB system (Supplementary Material, Appendix B). The MMB system uses a 532 nm laser diode module (Thorlabs, CPS532, Newton, NJ, USA), working at 0.25 mW. A dichroic mirror (Semrock, BrightLine Di02-R532, Rochester, NY, USA) diverts the excitation beam to an objective lens (Newport, M-10×, 0.25NA, Newport, CA, USA), which focuses the beam to a 150 µm spot size on a rectangular, borosilicate sample cell. The cell contains the fluorescently tagged beads and has inner dimensions of 8 mm × 0.4 mm × 70 mm (Vitrocom, W2540, Boonton, NJ, USA). The emitted fluorescence is collected using the same objective lens, passed through the dichroic mirror and two emission filters (Semrock, FF03-575/25, Rochester, NY, USA), and detected by a digital camera (FLIR, GS3-U3-23S6M-C, Wilsonville, OR, USA).

Two electromagnets, positioned 3 mm apart (one on each side of the glass sample cell), apply an alternating magnetic field gradient to the sample, causing the magnetic beads to aggregate and move together in a periodic lateral motion in and out of the laser beam. The modulation frequency of the electromagnets was set to 1 Hz. While the beads are moving, the laser beam remains fixed, causing the beads to form an “on” signal when they are in the laser beam and an “off” signal when they have moved out of the laser beam. The new electromagnets have parabolic pole tips (β= 0.85 mm^−1^) and the following parameters: L= 50 mm, w= 20 mm, D= 9 mm, h= 15 mm, d= 0.81 mm, c= 3 mm, and I= 1.4 A.

For each measurement, a sequence of 600 images is acquired over a period of 12 seconds. The grey value from the laser beam area of each image is integrated and the peak-to-peak voltage differences over time are calculated and averaged.

To validate the analytical performance of the new MMB system with the two small footprint electromagnets, we measured the same concentrations of recombinant ZIKV antibodies using a previous version of the MMB system. The standard MMB system, which was extensively analyzed [6,13,14,15,16], uses the relatively bulky and power consuming electromagnets. 

## 3. Results

### 3.1. Evaluating Different Pole Tip Shapes

**Figure 5:**
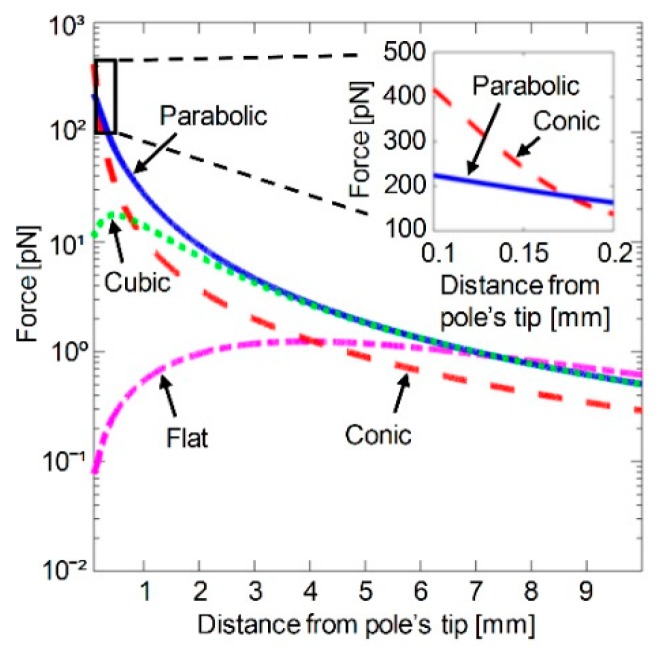
The magnetic force as a function of the distance from the pole’s tip for various magnetic pole tips. Inset: The magnetic force as a function of the distance from the pole’s tip for a conic pole tip and a parabolic pole tip.

Figure 5 presents the magnetic field force along the electromagnet’s symmetry axis for different pole tip shapes. At less than 0.17 mm from the pole tip, the highest magnetic force is generated by a conic pole tip. At a distance between 0.17 mm and 7.5 mm, the highest magnetic force is generated by a parabolic pole tip. At a distance over 7.5 mm from the pole’s tip, a flat-top pole tip generates a slightly higher magnetic force than any of the other pole tip shapes. At a relatively long distance from the pole tips (e.g., over 3 mm), the magnetic forces generated by a cubic pole tip and a parabolic pole tip are similar. However, at a relatively short distance (e.g., less than 3 mm), the magnetic force generated by a parabolic pole tip is up to 10-fold higher than that of a cubic pole tip (Figure 5). 

### 3.2. Characteristics of Magnetic Beads Manipulation and Mutual Magnetization in a Two-Electromagnet Setup

**Figure 6:**
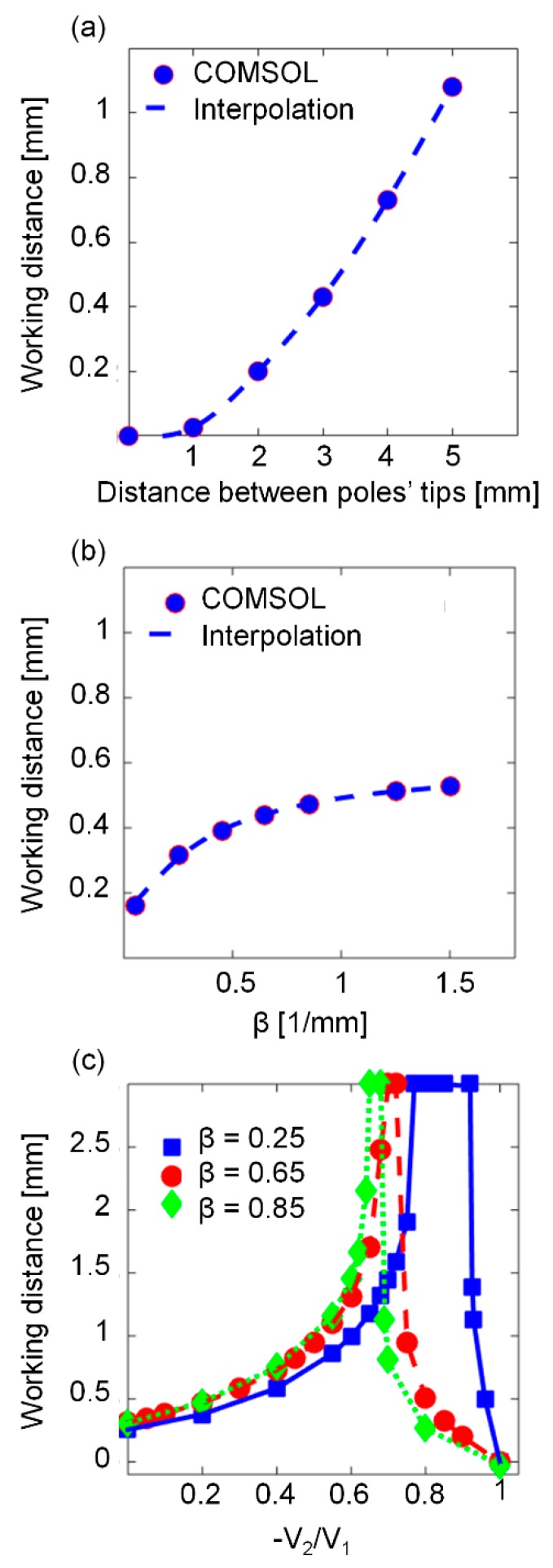
Characteristics of the working distance for a system of two electromagnets with parabolic pole tips that are positioned opposite each other. (**a**) The working distance as a function of the distance between the pole tips (β=0.65). (**b**) The working distance as a function of the parabolic coefficient, β (the distance between the pole tips is 3 mm). (**c**) The working distance as a function of the ratio between the voltages of the electromagnets (all β units are in [1/mm], and the distance between the pole tips is 3 mm).

Figure 6 depicts the characteristics of the working distance in a two-electromagnet system. The working distance significantly lengthens when the distance between the pole tips increases (Figure 6a). In addition, for a fixed distance between the pole tips, the working distance becomes larger when the parabolic coefficient, β, increases (Figure 6b). The broadening of the working distance as a function of the parabolic coefficient saturates at approximately β= 0.85.

The correlation between the working distance and the ratio between the voltages of the electromagnets is depicted in Figure 6c. When the voltage of the inactive electromagnet, $V_{2}$, is inverted (i.e., instead of 0 V during its inactive phase, it receives a negative voltage), the working distance can be broadened. The working distance reaches its maximum value (3 mm, the distance between the pole tips) when the ratio between the amplitudes of the inactive electromagnet and the active electromagnet is 0.6–0.9 (i.e., $-V_{2}/V_{1}=0.6–0.9$). The ratio for which the working distance reaches its maximum varies for different parabolic coefficients. When the ratio between the voltages increases further, the working distance drops until it becomes zero when the negative amplitude of the inactive electromagnet equals the positive amplitude of the active electromagnet (i.e., $-V_{2}/V_{1}=1$).

**Figure 7:**
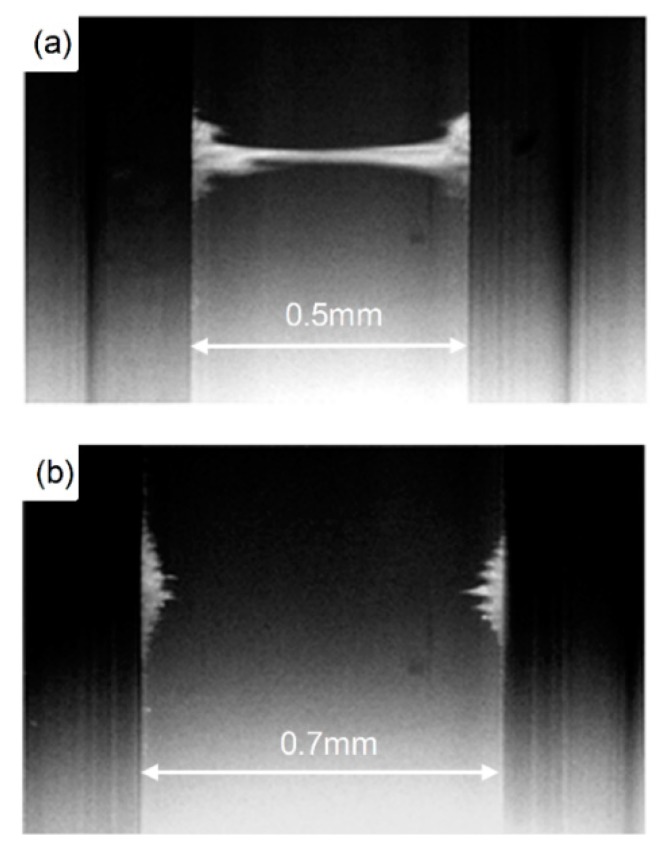
Experimental demonstration of the working distance in a system of two electromagnets with parabolic pole tips (β=0.65) that are positioned 4 mm opposite each other. (**a**) Magnetic beads can be manipulated from side to side in a 0.5 mm wide glass cuvette. (**b**) Magnetic beads cannot be manipulated from side to side in a 0.7 mm wide glass cuvette.

The correlation between the working distance and the distance between the pole tips was validated experimentally. When the distance between the two parabolic pole tips (β=0.65) was 4 mm, the beads could be manipulated inside a 0.5 mm wide cuvette (Figure 7a). However, when the inner width of the cuvette was 0.7 mm, the beads were trapped on the cuvette’s walls and could not be manipulated from side to side (Figure 7b). When the distance between the two parabolic pole tips was 3 mm, the beads could be manipulated inside a 0.4 mm wide cuvette, but not inside a 0.5 mm or wider cuvette (Supplementary Material, Movie S1). These experimental findings match well with the COMSOL Multiphysics® simulation results (Figure 6a).

**Figure 8:**
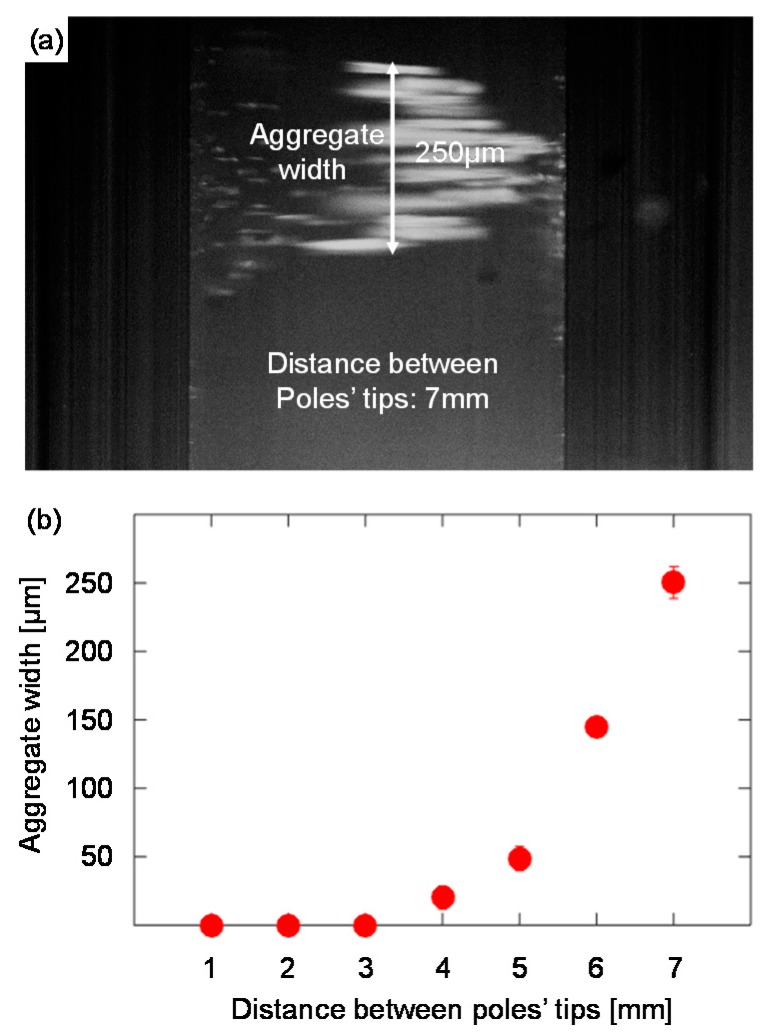
The correlation between the width of the aggregate and the distance between the pole tips. (**a**) The width of the beads’ aggregate when manipulated by two electromagnets with parabolic pole tips (β=0.65) that are positioned 7 mm opposite each other (the cuvette’s width is 0.6 mm). (**b**) Experimental measurements of the width of the aggregate as a function of the distance between the pole tips.

Figure 8 shows the correlation between the width of the beads’ aggregate and the distance between the pole tips. When the pole tips are located relatively far from each other (e.g., 7 mm apart), the beads move in a chain-shaped structure (Figure 8a). These swarms are not aggregated to form a cloud or a single aggregate. When the pole tips are closer to each other (e.g., 4 mm apart), the beads move as a single aggregate in a narrow line between the glass walls (Figure 7a). The width of the beads aggregate as a function of the distance between the pole tips is depicted in Figure 8b.

### 3.3. Optimizing Magnetic Forces in a Two-Electromagnet Setup

**Figure 9:**
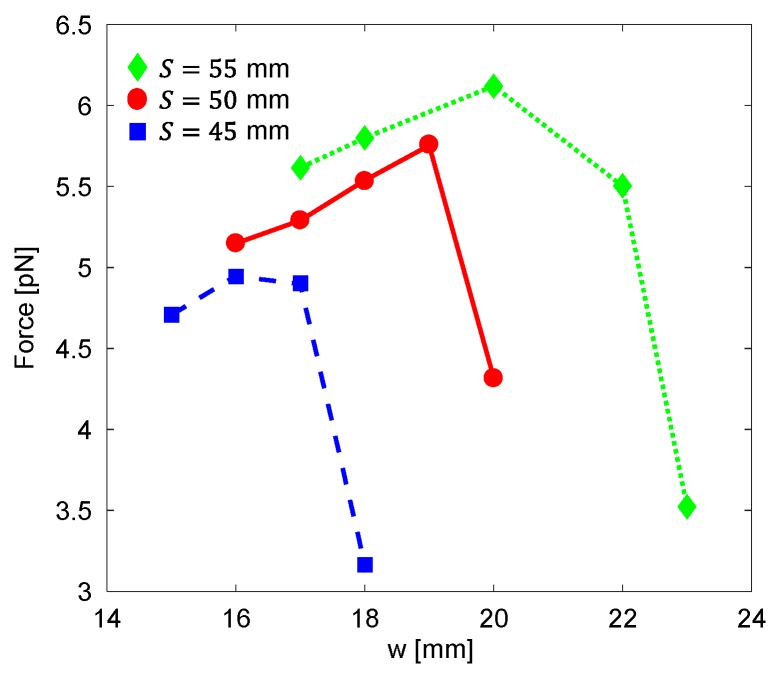
The magnetic force acting on a magnetic particle located at z=2 mm from an electromagnet with a parabolic pole tip as a function of the electromagnet’s winding area width. For a fixed total diameter of the electromagnet, S, the magnetic force is maximized by using the optimal combination of D, the pole’s diameter, and w, the winding area width. The core sleeve’s width is assumed to be 3 mm.

When the total diameter of the electromagnet is fixed (e.g., due to physical constraints of the system), there is a tradeoff between the winding area width, w, and the diameter of the pole, D. The magnetic force at z=2 mm as a function of the winding area width of electromagnets with different total diameters is depicted in Figure 9. For a fixed total diameter of the electromagnet, the magnetic force is maximized by using the optimal combination of the pole’s diameter and the winding area width. For example, when the total diameter of the electromagnet is 55 mm, the magnetic force is maximized for w=20 mm and D=9 mm (assuming c=3 mm). 

### 3.4. MMB IgM anti-Zika NS1 Dose Response Results

**Figure 10:**
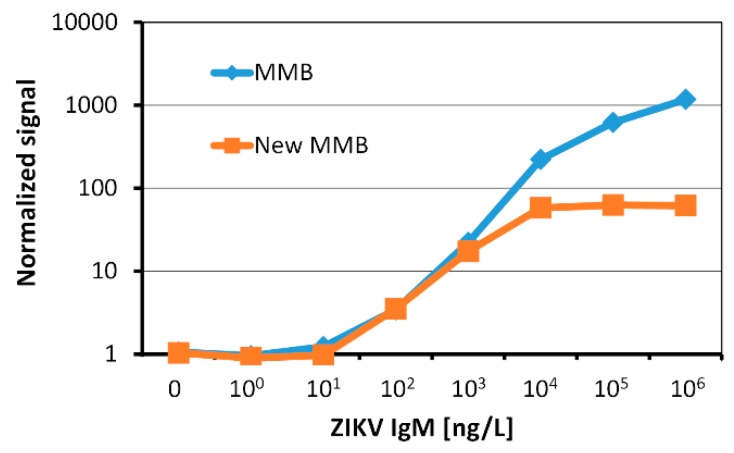
Dose responses of recombinant human IgM anti-Zika NS1 using a small footprint MMB system (“New MMB”) and a standard MMB system (“MMB”).

The dose response curves of the IgM antibodies in the small footprint MMB system (“New MMB”) and the standard MMB system (“MMB”) are depicted in Figure 10. The limits of detection, calculated as three standard deviations from the blank measurement, are 13 ng/L and 25 ng/L with dynamic ranges of 2-logs and 3-logs, respectively. 

## 4. Discussion

Efficient manipulation of magnetic beads heavily depends on the strength and structure of the magnetic field sources. Electromagnets provide strong and adjustable magnetic fields, and their lack of mechanical movement makes them less susceptible to mechanical malfunction than permanent magnets. Three key factors affect the performance of electromagnets: (1) the magnetic properties of the electromagnet’s pole material (e.g., magnetization saturation and maximum relative permeability), which affect the maximum magnetic field and the response time of the electromagnet, (2) the electromagnet’s physical parameters (e.g., winding area length and width, core diameter, wire diameter, current), and (3) the pole’s tip shape, which affects the strength and direction of the magnetic field near the pole tip. For example, the sharper the pole tip, the higher the magnetic field gradient near the tip. In applications that require high magnetic field gradients at very short distances from the pole tip (e.g., tens to a few hundred micrometers) [5,19], a conic pole tip is preferable. In applications that require high magnetic field gradients over a long range of distances from the pole tip (e.g., from a few hundred micrometers to several millimeters [14], a parabolic pole tip is preferred. At a long distance from the pole tip (e.g., inside blood vessels) [25], a flat-top pole tip, which is the easiest to produce, is sufficient.

When two or more electromagnets are used to manipulate magnetic beads in space, the mutual influence of the magnetic fields dictates the maximum working distance over which magnetic beads can be manipulated without being trapped by one of the electromagnets. To enable precise manipulation of magnetic beads over a large area, increasing the working distance is desirable. Here, we showed that, for parabolic pole tips, the working distance can be broadened by increasing the parabolic curvature. However, above a certain parabolic curvature (e.g., β=0.85), further broadening of the working distance is negligible. Significant broadening of the working distance (e.g., for applications that require transportation of magnetic beads over a long distance) can also be achieved by increasing the distance between the pole tips. Nevertheless, increasing the distance between the pole tips increases the total size of the device, reduces the magnetic field forces in both the z and r axas, and consequently modifies the shape of the beads’ aggregate. For example, when the parabolic pole tips are located away from each other, the magnetic beads move in chain-shaped clusters and do not form a cloud or a single aggregate. In some biosensing applications [6,14,15], the movement of the beads as a single aggregate is critical to achieving high repeatability between measurements. 

For a fixed distance between two identical electromagnets with parabolic pole tips, the working distance can be adjusted by changing the ratio between the maximum voltages of the two electromagnets. For example, when the distance between the poles is 3 mm and the parabolic curvature is β=0.65, the working distance reaches its maximum value (3 mm) if the ratio between the voltages of the electromagnets is $-V_{2}/V_{1}=0.7$.

In many lab-on-a-chip applications, physical constraints limit certain mechanical parameters, such as the length of the electromagnet, L, its total diameter, S, and the distance from the coil to the pole’s tip, h. When the total diameter of the electromagnet, S, and the core sleeve’s width, c, are fixed, there is a tradeoff between the winding area width, w, and the diameter of the core, D. Here, to minimize the size of an MMB system, we produced two electromagnets, each with a length of L=50 mm and a total diameter of S=55 mm. According to our simulation (Figure 9), the magnetic force is maximized for w=20 mm and D=9 mm (assuming c=3 mm). These electromagnets, which are approximately 21% smaller in volume than the previous version [6], produced sufficient magnetic force to aggregate magnetic beads and manipulate them from side to side in a small footprint MMB system. 

The analytical sensitivity of the new MMB system (13 ng/L) is on par with the standard MMB system. According to recent studies [16], this limit of detection enables detection of Zika IgM antibodies as early as 5 days and as late as 180 days post symptoms onset, significantly extending the number of days that the antibodies are detectable. It should be noted that the smaller dynamic range of the new MMB system is explained by the lack of additional neutral density filter before the camera. Thus, at a high concentration of target molecules, the strong fluorescent signal saturated the camera. This problem can be solved using a shorter exposure time whenever the camera saturates. 

## 5. Conclusions

Here, we provide a thorough analysis, including analytical calculations, numerical simulations, and experimental measurements, of using one or two electromagnets to manipulate magnetic beads. In general, the magnetic field gradient of an electromagnet, and subsequently the force acting on magnetic beads, can be significantly maximized for a specific application by carefully selecting the pole material and the tip’s shape. In addition, the shape of the beads’ aggregate and the distance over which they can be manipulated highly depends on both the position of the electromagnets relative to each other and their relative voltage.

In a two-electromagnet setup, to maximize the magnetic force acting on magnetic particles while maintaining the electromagnets’ minimal size and power consumption requirements, the following protocol can be used. First, based on power consumption constraints, select the maximum wire current, I, and the corresponding wire diameter, d. Second, based on the mechanical constrains, select the length of the electromagnet, L, its total diameter, S, and the distance from the coil to the pole’s tip, h. Third, to maximize the magnetic force, select the optimal pairing of the core diameter, D, and the winding area width, w. Then, based on the application and the distance between the pole tip and magnetic particles, select the pole material, shape, and curvature coefficient.

## Figures and Tables

**Figure A1:**
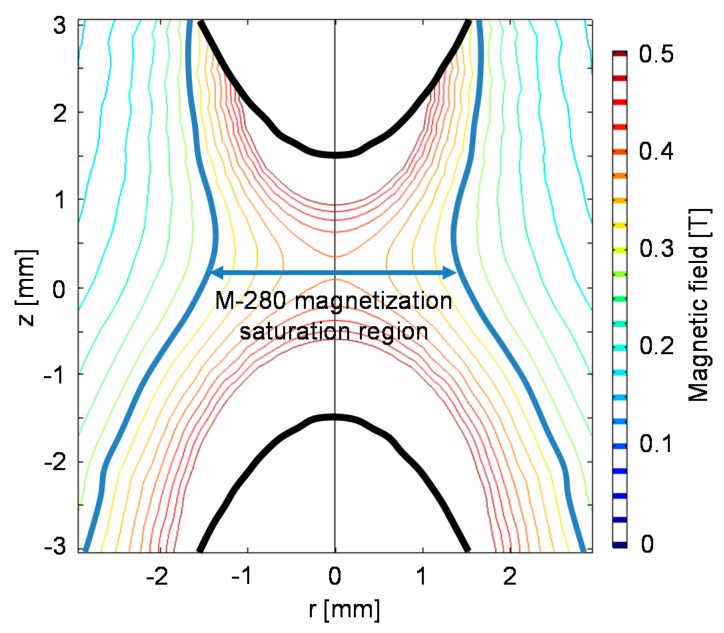
The area between two electromagnets in which the magnetic moment of the M-280 beads saturates. The spatial distribution of the magnetic field surrounding two electromagnets with parabolic pole tips (β=0.65) that are located 3 mm opposite each other. The contours represent the magnetic field magnitude. The magnetic moment of the M-280 beads saturates at an external field strength above 0.3 Tesla (marked with a thick blue line). Therefore, the magnetic moment of the magnetic beads saturates when the beads are located less than 2 mm from the pole tips.

**Figure A2:**
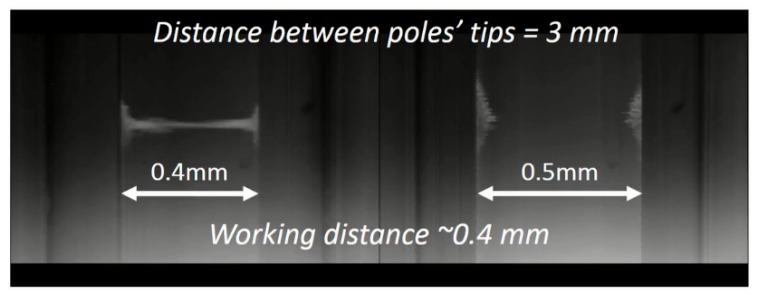
Movie S1. The correlation between the working distance and the distance between the pole tips. When the distance between the two parabolic pole tips (*β* = 0.65) is 3 mm, the working distance is ~0.4 mm, i.e., beads can be manipulated inside a 0.4 mm wide cuvette (left), but not inside a 0.5 mm or wider cuvette (right).

## Supplementary Materials

The following are available online at https://www.mdpi.com/2072-666X/10/11/784/s1, Figure S1: The area between two electromagnets in which the magnetic moment of the M-280 beads saturates. Movie S1: The correlation between the working distance and the distance between the pole tips.

## Appendix A

The magnetic force acting on a single magnetic bead, with a magnetic moment, $\vec{m}$, in an applied magnetic field, $\vec{B}$, is [26,27]:

(A1) $${\vec{F}}_{m}=\nabla \left(\vec{m}\left(\vec{B}\right)\cdot \vec{B}\right).$$

When the magnetic field is high, the magnetic moment saturates and becomes spatially constant:

(A2) $$\vec{m}=m_{s}\hat{B}$$

where $m_{s}$ is the magnetic bead’s saturation magnetization. Substituting Equation (A2) into Equation (A1) yields [28]:

(A3) $${\vec{F}}_{m}=m_{s}\hat{B}\cdot \nabla \vec{B}.$$

The magnetic field gradient can be represented as a tensor:

(A4) $$\nabla \vec{B}=\left(\begin{array}{ccc} \partial_{r}B_{r} & \partial_{r}B_{\phi } & \partial_{r}B_{z}\\ \frac{1}{r}\partial_{\phi }B_{r} & \frac{1}{r}\partial_{\phi }B_{\phi } & \frac{1}{r}\partial_{\phi }B_{z}\\ \partial_{z}B_{r} & \partial_{z}B_{\phi } & \partial_{z}B_{z} \end{array}\right).$$

When the magnetic field is applied using an electromagnet with a cylindrical symmetry, the tensor can be reduced to:

(A5) $$\nabla \vec{B}=\left(\begin{array}{cc} \partial_{r}B_{r} & \partial_{r}B_{z}\\ \partial_{z}B_{r} & \partial_{z}B_{z} \end{array}\right).$$

Substituting Equation (A6) into Equation (A3) yields:

(A6) $${\vec{F}}_{m}=m_{s}\hat{B}\cdot \left(\begin{array}{cc} \partial_{r}B_{r} & \partial_{r}B_{z}\\ \partial_{z}B_{r} & \partial_{z}B_{z} \end{array}\right)=\left(m_{s}/\left|\vec{B}\right|\right)\cdot \left(\begin{array}{cc} B_{r}\partial_{r}B_{r}+B_{z}\partial_{r}B_{z} & B_{r}\partial_{z}B_{r}+B_{z}\partial_{z}B_{z} \end{array}\right).$$

Hence, the magnitude of the magnetic field is:

(A7) $$F_{m}=\left|{\vec{F}}_{m}\right|=\left(m_{s}/\left|\vec{B}\right|\right)\cdot {\left({\left(B_{r}\partial_{r}B_{r}+B_{z}\partial_{r}B_{z}\right)}^{2}+{\left(B_{r}\partial_{z}B_{r}+B_{z}\partial_{z}B_{z}\right)}^{2}\right)}^{1/2}.$$

## Appendix B

Each MMB IgM anti-Zika assay consisted of ~25,000 tosylactivated magnetic beads (M-280, Thermo Fisher Scientific, Waltham, MA, USA), which were pre-conjugated to the Zika NS1-suriname strain (The Native Antigen Company, UK) using Thermo Fishers’ standard coupling procedures. The beads were incubated for one hour at room temperature with human IgM anti Zika NS1 in concentrations of 0, 10^1^, 10^2^, 10^3^, 10^4^, 10^5^, 10^6^, and 10^7^ ng/L. The initial incubation was followed by incubation with a fluorescently labeled detection antibody (Anti-Human IgM (µ-chain specific)-Biotin, Sigma–Aldrich Ltd., Israel). After a second wash, the beads were incubated with R-Phycoerythrin-Streptavidin (The Jackson Lab., USA). To remove unbound detection antibodies, a single buffer replacement was done after the final incubation. The final solution was then loaded into a borosilicate glass cuvette and measured in the MMB system. The number of repetitions of the blank measurement was six (n=6). The number of repetitions for all other concentrations was three (n=3). The limit of detection (LoD) for each set of experiments was calculated as three standard deviations over the blank measurement. The reaction buffer solution was a mixture of phosphate buffered saline (PBS ×1), 1 mg/ml of bovine serum albumin (VWR, Rodnor, PA, USA), and 0.05% of Tween-20 (Sigma–Aldrich, St. Louis, MO, USA).
